# Assessment-driven selection and adaptation of exercise difficulty in robot-assisted therapy: a pilot study with a hand rehabilitation robot

**DOI:** 10.1186/1743-0003-11-154

**Published:** 2014-11-15

**Authors:** Jean-Claude Metzger, Olivier Lambercy, Antonella Califfi, Daria Dinacci, Claudio Petrillo, Paolo Rossi, Fabio M Conti, Roger Gassert

**Affiliations:** Rehabilitation Engineering Laboratory, ETH Zurich, Leonhardstrasse 27, 8092 Zurich, Switzerland; Clinica Hildebrand Centro di Riabilitazione Brissago, Via Crodolo, 6614 Brissago Switzerland

**Keywords:** Patient-tailored, Customized, Individualized, Neurocognitive therapy, Sensory function, Proprioception, Hand function, Patient-cooperative

## Abstract

**Background:**

Selecting and maintaining an engaging and challenging training difficulty level in robot-assisted stroke rehabilitation remains an open challenge. Despite the ability of robotic systems to provide objective and accurate measures of function and performance, the selection and adaptation of exercise difficulty levels is typically left to the experience of the supervising therapist.

**Methods:**

We introduce a patient-tailored and adaptive robot-assisted therapy concept to optimally challenge patients from the very first session and throughout therapy progress. The concept is evaluated within a four-week pilot study in six subacute stroke patients performing robot-assisted rehabilitation of hand function. Robotic assessments of both motor and sensory impairments of hand function conducted prior to the therapy are used to adjust exercise parameters and customize difficulty levels. During therapy progression, an automated routine adapts difficulty levels from session to session to maintain patients’ performance around a target level of 70%, to optimally balance motivation and challenge.

**Results:**

Robotic assessments suggested large differences in patients’ sensorimotor abilities that are not captured by clinical assessments. Exercise customization based on these assessments resulted in an average initial exercise performance around 70% (62% ± 20%, mean ± std), which was maintained throughout the course of the therapy (64% ± 21%). Patients showed reduction in both motor and sensory impairments compared to baseline as measured by clinical and robotic assessments. The progress in difficulty levels correlated with improvements in a clinical impairment scale (Fugl-Meyer Assessment) (r _*s*_ = 0.70), suggesting that the proposed therapy was effective at reducing sensorimotor impairment.

**Conclusions:**

Initial robotic assessments combined with progressive difficulty adaptation have the potential to automatically tailor robot-assisted rehabilitation to the individual patient. This results in optimal challenge and engagement of the patient, may facilitate sensorimotor recovery after neurological injury, and has implications for unsupervised robot-assisted therapy in the clinic and home environment.

**Trial registration:** ClinicalTrials.gov, NCT02096445

**Electronic supplementary material:**

The online version of this article (doi:10.1186/1743-0003-11-154) contains supplementary material, which is available to authorized users.

## Background

Active participation is known to be a key parameter that influences the outcome of rehabilitation therapy in stroke survivors [1–4]. To maximize engagement during therapy and prevent frustration, it is essential to design rehabilitation exercises in such a way that they challenge patients at a difficulty level in which exercises are neither too simple, nor too difficult [5, 6]. Furthermore, motor learning studies have shown that matching the difficulty of a task to the learner’s initial skill level, and further adapting it as learning progresses, enhances learning efficacy [7]. In clinical settings, the selection of exercise difficulty and its adaptation over the course of a therapy is a challenging task, often left to the experience of trained therapists and their subjective perception of a patient’s abilities [8].

The emergence of robotic devices and virtual reality environments to complement conventional rehabilitation has opened up new perspectives for the automatic selection and on-line adaptation of therapy difficulty [9]. In addition to motivating and well-controlled exercises, robotic devices can provide objective and accurate assessments of function and impairment [10]. They offer the possibility to continuously monitor patient performance, and correspondingly adapt therapy intensity and difficulty after each session or even each trial, in a way that optimally challenges patients throughout the therapy [11].

Several strategies have been proposed for adapting therapy difficulty to a patient’s individual needs and/or abilities during robot-assisted rehabilitation. A common approach consists in varying the amount of assistance a robotic system can provide to the patient, in an “assist-as-needed” fashion [12]. The assistance provided to complete a specific task is progressively decreased (per session, or even online), thereby increasing task difficulty to optimally engage and challenge patients. Assistance modulation can be based on the patients’ active participation and performance in the task, as measured by interaction forces, muscle activity, or other kinematic or physiological parameters [13–16]. However, increasing the level of robotic assistance in case of poor performance/participation could eventually lead to slacking, where patients progressively become passive (decrease their level of muscle activation and thus active contribution) and rely on the assistance of the robot, despite being able to actively participate in the task [17, 18].

Another approach to adjusting therapy difficulty relies on modifying spatiotemporal parameters of the task to be achieved during a rehabilitation exercise without changing the level of robotic support/guidance. This could take the form of progressively more complex movement patterns to be achieved [11, 19], more distant positions to be reached [11, 20–23], a smaller time window to complete a task [21, 24], or increased interaction force required from the patient [6, 23, 25, 26]. Typically, different pre-determined levels of increasing difficulty are implemented, similar to video games, and patients navigate from level to level (on a session or even trial basis) according to performance scores computed from kinematic or dynamic metrics.

However, if an exercise is started at a default difficulty level, i.e. the same difficulty level is selected for all patients despite their diverse impairments, exercises might be too difficult or too simple for an individual patient, and several therapy trials/sessions might be necessary to adapt the exercise to the difficulty level which is appropriate. One way to solve this issue consists in performing initial robotic assessments, prior to therapy start, to identify each patient’s ability to perform a specific task with the robot, and subsequently adapting the initial task difficulty throughout the therapy [21, 22, 24].

In this paper, we present a novel therapy adaptation approach combining (i) initial robotic assessments to establish sets of patient-specific difficulty levels for therapy exercises as well as to select initial exercise difficulty, and (ii) subsequent session-wise challenge adaptation throughout the course of a therapy, by automatically progressing through the patient-specific difficulty levels based on daily exercise performance. As a proof of concept, this approach was clinically evaluated in the context of a four-week pilot study on neurocognitive robot-assisted therapy of hand function with the ReHapticKnob, a 2 degrees-of-freedom (DOF) robotic device [27, 28]. For the initial robotic assessments, both motor and sensory function of the hand were evaluated prior to the start of the therapy, by evaluating active range of motion in hand opening and forearm pronation/supination, and smallest perceptible differences in distance (finger aperture) and stiffness (object grasping). Outcome measures from the robotic assessments were then used to define patient-specific difficulty levels for a set of seven neurocognitive rehabilitation exercises implemented on the ReHapticKnob. During the therapy sessions, a simple algorithm aimed at adjusting the difficulty level to maintain patients’ performance in each exercise at a success rate of 70%, which is often referred to as an optimal balance between motivation and challenge [6, 20, 21]. We hypothesized that the combination of initial assessment-driven difficulty selection and subsequent performance-based progression in difficulty levels would allow to appropriately challenge patients from the very beginning of the therapy and maintain this challenge over therapy sessions while impairment levels are expected to decrease. Within the limits of device capabilities, this approach should generalize to most rehabilitation robots.

## Methods

### Participants

Six subacute stroke patients (72.8 ± 12.0 years old (mean ± std)) were enrolled in this pilot study. Inclusion criteria included a hemiparesis caused by a first occurrence of stroke (<6 weeks) and age between 18 and 90 years. Exclusion criteria comprised an altered state of consciousness, severe aphasia (Goodglass and Kaplan test < 1, [29]), severe cognitive deficits (Level of Cognitive Function-Revised <6, (Hagen C: Level of Cognitive Functioning-Revised, unpublished)), severe pain syndrome (Visual Analog Scale ≥5, [30]), or severe pathologies of the upper extremity of traumatic or rheumatic origin.

All participants showed mild upper limb impairment (56.0 ±3.7 on the Fugl-Meyer Assessment (FMA-UE) [31]) resulting from ischemic stroke. Prior to being enrolled, all patients were informed about the study and any related potential risks, and they provided written consent. The study was approved by the ethics commission of the Canton of Ticino (Ref. CE 2646) and is registered at ClinicalTrials.gov, NCT02096445. Table 1 details the patient demographics.

**Table 1:** **Patient demographics**

Patient	Age [years]	Gender	Handed -ness	Impaired hand	Post lesion [weeks]	Initial FMA-UE^1^	Initial FMA^1^subscore(hand/ wrist)	Neurological disorder
P1	85	F	R	L	2	57	20	Ischemic stroke in the right corona radiata and frontal centrum semiovale
P2	67	M	R	L	2	55	20	Ischemic stroke in right thalamus
P3	80	M	R	L	5	59	19	Ischemic stroke in right ponto-cerebellar region
P4	70	M	R	R	6	52	16	Ischemic stroke in left parietal lobe
P5	53	M	R	L	4	52	17	Ischemic stroke in right pre and post-central gyrus and right parietal lobe
P6	82	M	R	L	3	61	20	Ischemic stroke in cortico-subcortical temporal-parietal lobe
Mean	72.8	-	-	-	3.7	56.0	18.7	-
(Std)	(12.0)	-	-	-	(1.5)	(3.7)	(1.8)	-

^1^Fugl-Meyer Assessment (FMA) [31]. FMA scores for the upper extremity (maximum score = 66) and hand/wrist (maximum score = 24) subsections are reported (lower scores indicate greater impairment).

### Study protocol

**Figure 1:**
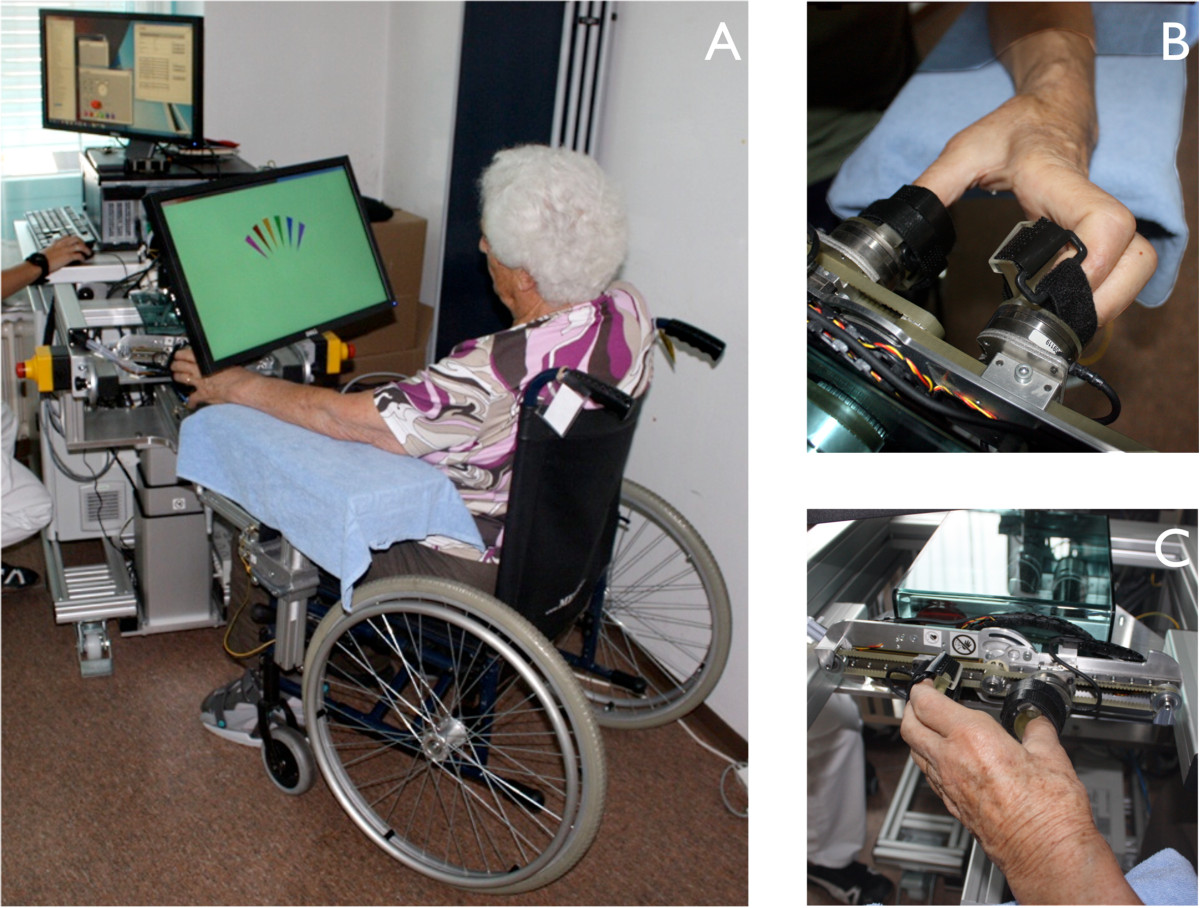
**Neurocognitive therapy with the ReHapticKnob.** **A**: direct vision of the hand is blocked through the placement of the computer monitor over the patient’s hand. The screen shows information relevant to the execution of the respective exercise. **B** and **C**: thumb and fingers are attached to the finger supports of the ReHapticKnob and held in place with Velcro straps.

The proposed assessment-driven selection and adaptation of therapy difficulty was implemented within a four-week pilot study on neurocognitive robot-assisted therapy of hand function carried out at the Clinica Hildebrand Centro di Riabilitazione Brissago in Switzerland. This study was performed with the ReHapticKnob, a two DOF hand rehabilitation robot to train grasping and/or forearm rotation (pronosupination of the forearm) (Figure 1) [27]. The ReHapticKnob is based on an end-effector design with exchangeable finger supports (e.g. a small support for the thumb and a large support for the opposing fingers as used in this study) to accommodate different hand sizes. Fingers can be fixed to the supports with Velcro straps. In addition to standard safety mechanisms (emergency button and mechanical end-stops), implemented software limits on grasping aperture (respectively forearm rotation) and interaction force (respectively torque) guarantee the safe use of the device [27]. A physiotherapist assured that patients were able to perform the implemented exercises without overstretching or pain.

On four days per week, patients received a 45 minute session of neurocognitive robot-assisted therapy with the ReHapticKnob. Neurocognitive therapy follows the concepts developed by Perfetti [32], and trains patients in solving cognitive tasks through physical interaction with their environment (e.g. object exploration and identification). Exercises typically require patients to rely on tactile and/or proprioceptive feedback from their impaired limb following active or passively guided movements. The implementation of neurocognitive exercises on a robotic device is particularly suitable, as robots can render a large variety of haptic cues to simulate physical interaction with the environment [28].

**Figure 2:**
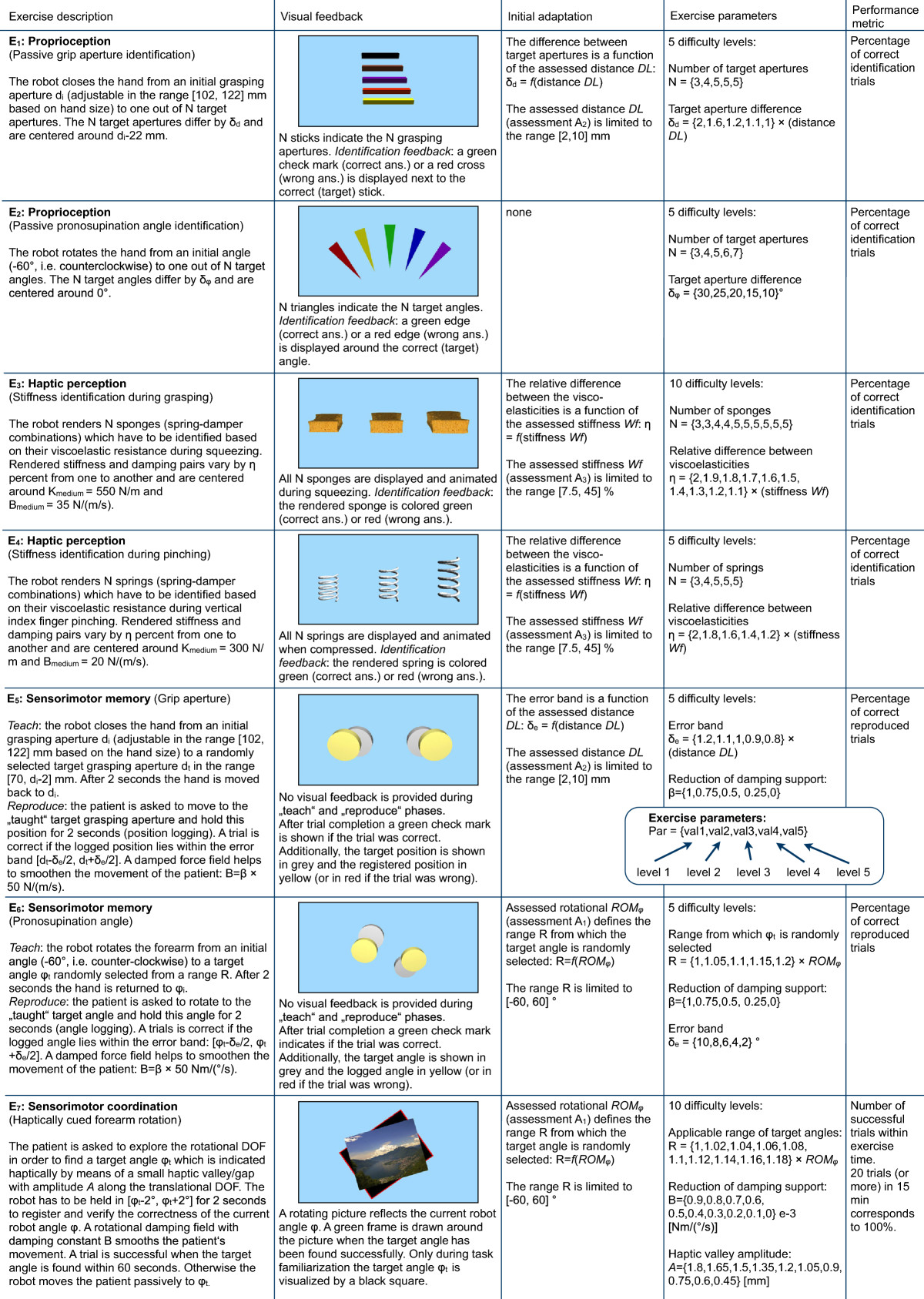
**Detailed description of exercises** ***E***_***1***_**-** ***E***_***7***_**.** The heuristically defined exercise parameters used to customize the difficulty levels are shown within curly brackets in the “Exercise parameters” column. Refer to the flowchart in Figure 3 for a description of the patient-tailored and adaptive therapy concept.

Each therapy session with the robot was composed of a subset of three exercises lasting up to 15 minutes each and conducted in a randomized order. The three exercises trained in each session were selected among seven neurocognitive exercises, *E*_1_- *E*_7_, implemented on the robot and focusing on important aspects of hand sensorimotor function (i.e. proprioception, haptic perception, sensorimotor memory and sensorimotor coordination; refer to Section on Neurocognitive robot-assisted rehabilitation and Figure 2). An exercise plan for all therapy sessions, common to all participants, was defined prior to the study start in order to ensure that each exercise was regularly trained.

In parallel to the robot-assisted therapy sessions, patients followed the usual daily rehabilitation program for subacute inpatients at the Clinica Hildebrand Centro di Riabilitazione Brissago. This consisted of a 45 minute session of conventional neurocognitive therapy without the robot, as well as additional therapy sessions not focused on the upper limb, but which could nevertheless also involve upper extremity exercises, e.g. during physiotherapy or occupational therapy.

To evaluate and monitor upper limb impairment, clinical (FMA-UE) and robotic assessments were conducted at three time points and on separate days from the therapy: before (*pre*) and after the four weeks of the study (*post*), as well as in an additional follow-up assessment four weeks after the completion of the robot-assisted therapy (*follow-up*).

### Robotic assessments

Three robotic assessments (*A*_1_- *A*_3_) were implemented on the ReHapticKnob with the aim of evaluating the active range of motion while manipulating the finger supports, as well as proprioception and haptic perception. Data from the initial robotic assessments were used to establish patient-specific difficulty levels and to select the initial difficulty for each exercise. These assessments are described in the following.

#### ***A***_**1**_***- Range of motion (ROM***_***φ***_***and ROM***_***x***_***)***

The active range of motion in pronation/supination on the robot was assessed by asking the patient to rotate the end-effector to the maximum reachable pronation angle *φ*_*pmax*_, followed by the maximum supination angle *φ*_*smax*_. Similarly, the active range of motion in grasping was measured as patients moved from their minimal grip aperture on the robot *x*_*min*_ to their maximal grip aperture *x*_*max*_. The range of motion along these two DOF was defined as:

1 $${\text{ROM}}_{\varphi }=\varphi_{p\;\text{max}}-\varphi_{s\;\text{max}}$$

2 $${\text{ROM}}_{x}=x_{\text{max}}-x_{\text{min}}$$

#### ***A***_**2**_***- Proprioception***

Hand proprioception was assessed by measuring the just perceptible distance difference threshold, respectively difference limen (distance *DL*), at 80 mm grasping aperture using methods from psychophysics. A two-alternative forced choice (2AFC) paradigm was used, which consists in consecutively and randomly presenting two different stimuli, a standard stimulus *St* and a comparison stimulus *Co*, after which patients were asked to indicate which of the two stimuli was perceived as the larger [33]. The 2AFC paradigm has been selected as it is expected to be more objective and almost bias-free compared to other psychophysical paradigms [34].

In the case of the proprioception assessment, the robot passively opened the patient’s hand from an initial grasping aperture of 62 mm, selected as a suitable grip aperture for most patients, to a standard grip aperture *St* = 80 mm or to a comparison aperture *Co*=*St*+*Δd*. It has been shown that the distance DL is dependent on hand position, i.e. grip aperture [35]. Hence, assessment *A*_2_ was conducted at a standard initial grip aperture, corresponding to the position later used in the robotic exercises, such that the exercise difficulty could be appropriately adjusted. An initial stimulus difference *Δd*= 6 mm was chosen and adjusted adaptively using parameter estimation by sequential testing (PEST). This method was selected for its fast and accurate algorithm convergence [36]. Based on the level of correct stimulus identification of the patient, PEST specifies a set of heuristic rules which define if the previously tested stimulus difference *Δd* should be kept constant, respectively increased or decreased by an adaptive step *s*. The selectable PEST parameters were chosen such that the algorithm converged to a *Δd* where patients provided 75% correct answers. Convergence to the smallest perceptible distance *DL* was achieved when 15 consecutive trials at the same stimulus difference were executed, or if the step *s* fell below a predefined minimum step *σ*. If the algorithm did not converge, the assessment was terminated after 20 minutes to prevent fatigue, and the distance *DL* was set to the last tested *Δd*. For the proprioception assessment, an initial step *s* = 2 mm and a minimum step *σ* = 0.5 mm were selected based on good convergence during test runs with healthy subjects.

#### ***A***_**3**_***- Haptic perception***

A similar approach as for the proprioception assessment was used to evaluate patients’ ability to perceive and differentiate haptic stimuli during active object grasping. Two virtual springs, with standard stiffness *St* and comparison stiffness *Co* = *St* · (100*%* + *Δk*_*%*_) were rendered by the robot. The ability to discriminate stiffness is affected by the patient’s conscious or unconscious discrimination strategy, which might favor force and/or position cues [37]. Further, the smallest detectable stimulus differences for stiffness follow Weber’s law, i.e. the detectable difference varies relative to the tested standard stimulus [37]. Hence, the assessed (relative) stiffness difference *Δk*_*%*_ was represented in percentages of the standard stiffness *St* = 300 N/m and converged to the patients’ stiffness Weber fraction (stiffness *Wf*). An initial stimulus difference *Δk*_*%*_= 35 *%*, an initial step *s* = 10% and a minimum step *σ*= 2.5 *%* were empirically selected based on prior tests with healthy subjects.

### Neurocognitive robot-assisted exercises

Seven exercises *E*_1_- *E*_7_ (briefly described in this section and in more detail in Figure 2) motivated by conventional neurocognitive exercises [32] were implemented on the ReHapticKnob. The exercises train four key concepts; (i) proprioception, (ii) haptic perception, (iii) sensorimotor memory, and (iv) sensorimotor coordination. All exercises involve solving a cognitive task based on sensory inputs from the hand. Vision of the tested hand is obstructed in all exercises by a computer monitor placed over the hand, which is further used to provide instructions and feedback related to each exercise (see Figure 1).

#### ***E***_**1**_***& E***_**2**_***- Proprioception***

Patients are asked to identify different grip apertures (*E*_1_) or different pronosupination angles (*E*_2_) to which their hand is passively moved by the robot. Patients verbally report the perceived stimulus by selecting one of the possible positions shown on the monitor. To familiarize with the tasks and memorize the haptic stimuli, up to ten test trials (not used for exercise performance estimation as elaborated below) with visual feedback of the presented aperture were provided in each session. These passive exercises are thought to be feasible even by severely impaired patients without requiring assistance from supervising physiotherapists.

#### ***E***_**3**_***& E***_**4**_***- Haptic perception***

Patients are asked to actively grasp the end-effector of the robot to identify different viscoelastic force fields representing virtual sponges rendered by the robot and displayed on the monitor (*E*_3_). In *E*_4_ a similar concept is trained, where the end-effector of the robot is rotated by 90° so that patients identify different virtual springs by vertically pressing down on the springs with their index finger. Similarly to *E*_1_ and *E*_2_, test trials are also provided in each session, where visual feedback on the presented stimulus is provided on the monitor.

#### ***E***_**5**_***& E***_**6**_***- Sensorimotor memory***

In a first phase of this exercise, the patient’s hand is passively moved from a starting position to a target grip aperture (*E*_5_) or to a target pronosupination angle (*E*_6_), and held there for 2 seconds before returning to the start position. Details on the target selection process can be found in Figure 2. In the second phase, patients are asked to actively reproduce the movement by displacing the finger supports to the same target position/angle, and hold this position/angle for 2 seconds within a specific position error window.

#### ***E***_**7**_***- Sensorimotor coordination***

Patients are asked to actively pronate/supinate their forearm to reach a target angle indicated haptically by a small valley/gap along the translational DOF (see [28] for additional details). This exercise requires patients to combine and coordinate sensory feedback from the fingers while actively performing position exploration with the forearm.

In the elaboration of the therapy plan, it was decided to perform more sessions with exercises *E*_3_ and *E*_7_, setting a focus on compliance identification during grasping as well as the coupled training of forearm rotation and grasping, which were identified as the exercises corresponding best to daily activities. To maximize participation in the exercises, physiotherapists could assist patients’ movements or prevent their hand from slipping off the finger supports if patients could not execute the movements on their own, but were requested not to assist patients in the cognitive tasks related to each exercise.

### Initial selection and automatic adaptation of difficulty levels

**Figure 3:**
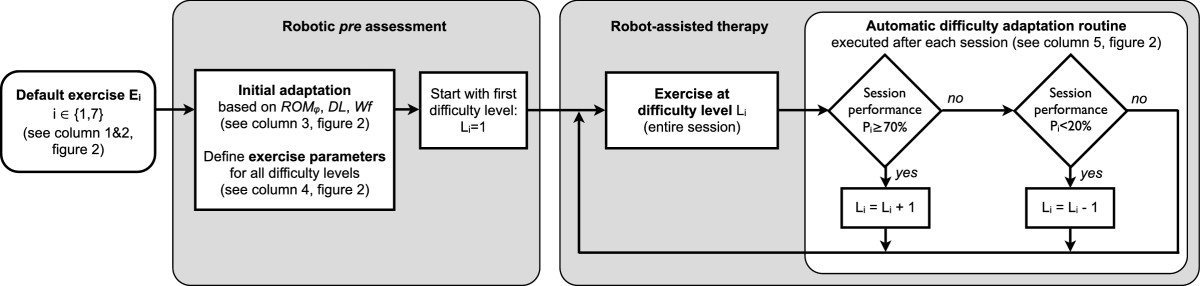
**Patient-tailored and adaptive therapy concept.** The difficulty levels of the neurocognitive robot-assisted exercises (detailed in Figure 2) are customized before the therapy onset using the assessed rotational range of motion (*ROM*_*φ*_), the just perceptible difference threshold in grasping aperture (*DL*) and the stiffness Weber fraction (*Wf*). An automatic difficulty adaptation routine adjusts the exercise difficulty level on a session-by-session basis according to the performance during the last session of the respective exercise (performance computed over the entire exercise session).

Both an initial and an automatic difficulty adaptation were used to individualize and adapt the robotic therapy in an attempt to optimally challenge patients throughout the entire therapy, targeting an exercise performance of *P* = 70% starting from the very first therapy session (Figure 3).

Before the robotic therapy sessions were started, exercise parameters, such as movement amplitude or magnitude difference between haptic stimuli (see Figures 2 and 3), were selected to adapt the exercise parameters of the initial difficulty level *L*_*i*_= 1 (*i* ∈ {1,7}) to initial functional ability of each individual patient according according to the outcomes of robotic *pre* assessments (*ROM*_*φ*_, *DL* and *Wf*). Based on this initial difficulty level, more advanced difficulty levels were computed by incrementally changing exercise parameters with respect to those of the initial level (Figure 3). Heuristic parameter increments were scaled with the outcomes of the individual *pre* assessments (*ROM*_*φ*_, *DL*, *Wf*). Therefore, all difficulty levels were “tailored” with respect to the initial ability of the patient, as explained in detail in Figure 2. As an example of how to interpret Figure 2, a patient assessed with a distance *DL* of 4.25 mm during *A*_2_ will start exercise *E*_1_ with an initial difficulty level (*L*_1_ = 1) with N = 3 different grasping apertures to discriminate, i.e. 71.5 mm, 80 mm and 88.5 mm. These grasping apertures differ by *δ*_*d*_ = 2 · distance *DL* = 8.5 mm, and are centered around *d*_*i*_-22 = 80 mm, where *d*_*i*_= 102 mm corresponds to a comfortable aperture for the patient’s hand on the finger supports (depending on hand size). In the second difficulty level (*L*_1_ = 2), 4 grasping apertures are presented, differing by *δ*_*d*_ = 1.6 · distance *DL* = 6.8 mm, and again centered around d _*i*_-22 = 80 mm, i.e., 69.8, 76.6, 83.4 and 90.2 mm.

The progression from one level of difficulty to the next was ruled by an automatic difficulty adaptation routine, which updates the difficulty level from session to session based on the patient’s performance in the last therapy session *P*_*i*_ of an exercise *E*_*i*_:

$$L_{i}=\left\{\begin{array}{ll} L_{i}+1 & ,\text{if}\;P_{i}\geq 70\%\\ L_{i} & ,\text{if}\;P_{i}\in \;]20,70[\;\%\\ L_{i}-1 & ,\text{if}\;P_{i}\leq 20\% \end{array}\right.$$

where *i* ∈ {1,7}. Performance in an exercise was evaluated by the percentage of successfully completed trials, i.e. correctly identified stimuli or properly reproduced apertures/orientations. The selection of difficulty adaptation thresholds was motivated by the work of other groups [6, 20, 21], and by observations form our previous work [25].

## Results

**Figure 4:**
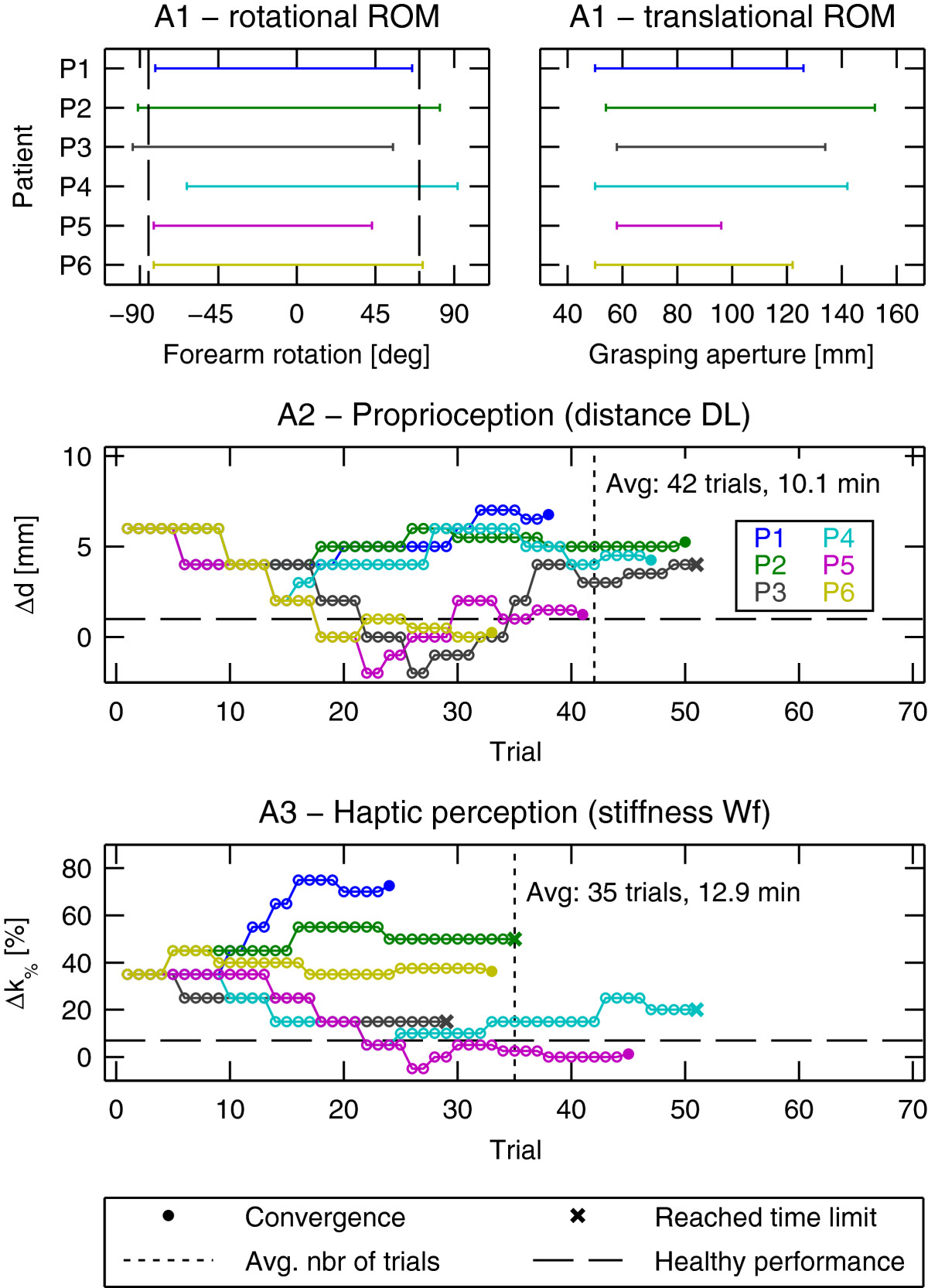
**Results of the robotic assessments A1-A3 during the** ***pre*** **assessment (week 0).** *Top:* pronosupination (left) and grasping aperture (right) range of motion. *Bottom:* Patient-wise evolution of the presented stimulus differences *Δ*d and *Δ* *k*_*%*_ to assess proprioception during hand opening/closing and haptic perception during grasping. Presented stimulus levels are adaptively selected by the PEST algorithm and converge to the smallest perceptible difference. Four assessment runs did not converge within the predefined time constraint of 20 minutes and are indicated with a cross (x) for the last trial. Healthy performance is indicated with dashed lines for comparison (forearm pronation 70°, forearm supination -85° [38], distance *DL* = 1 mm [35], stiffness *WF* = 7 *%*[37]), except for translational ROM, which depends on the hand size.

**Figure 5:**
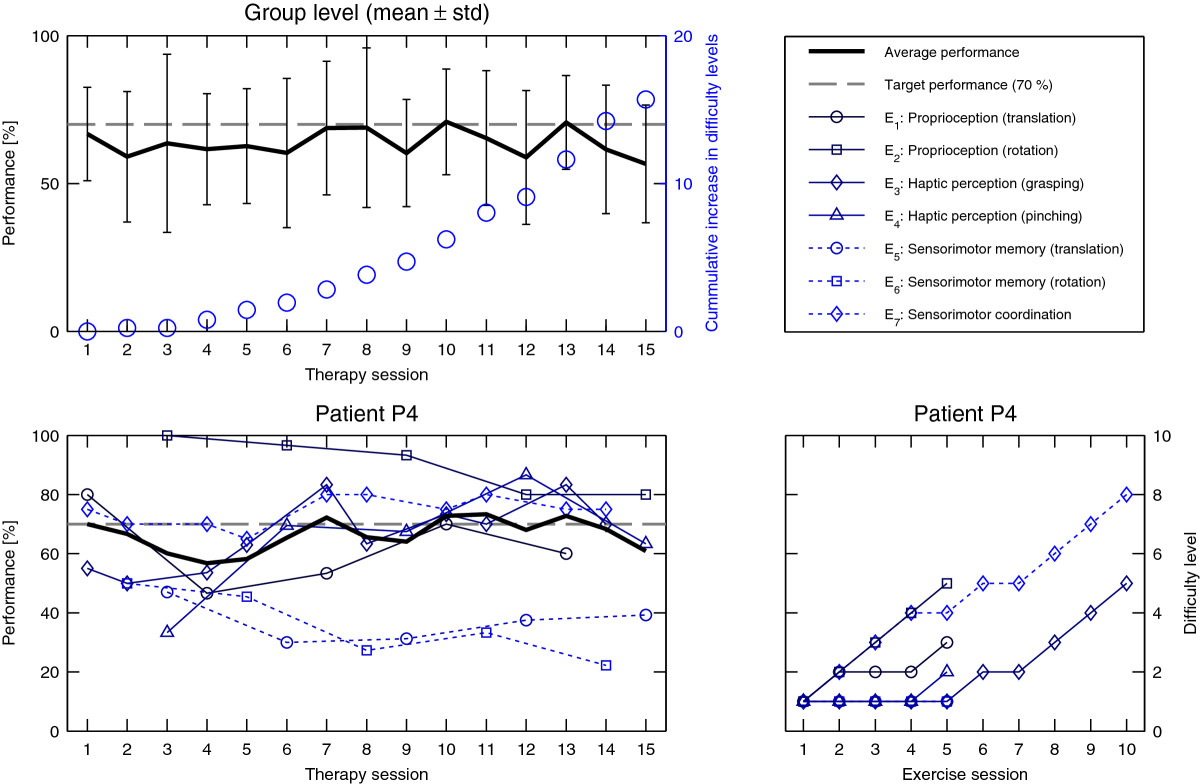
**Therapy exercise performance.** *Top:* Average therapy session performance (mean and standard deviation over all patients) tracks the desired 70% level (left y-axis). During therapy progression, difficulty levels (averaged over all patients) continuously increase (right y-axis, blue circles). *Bottom:* Exercise-wise performance evolution and corresponding difficulty level adjustments for a representative patient (P4). Note that only a subset of 3 exercises was performed during each therapy session.

All six subacute stroke patients were able to participate in the study, completing all of the robotic assessments and therapy sessions. In the first robotic assessment, patients exhibited different performance levels, as shown in Figure 4, suggesting different initial impairment levels. Overall, patients typically showed reduced rotational range of motion compared to healthy behavior (on average -2.9%, [38]), larger smallest perceptible distance difference threshold *DL* (on average +260%, [35]), and larger stiffness Weber fractions *Wf* (on average +364%, [37]).The outcome of the first robotic assessment was used to define the initial difficulty level of the seven exercises in order to offer challenging tasks from the onset of therapy. Averaged over all patients and exercises, the initial performance level, measured as the percentage of successfully achieved trials in the first occurrence of each exercise, was 62% ±20% (mean ±std). The difficulty progression algorithm developed in this work was able to maintain the average performance of a session (at the group level) around the desired 70% (64% on average over all sessions, minimum 57% and maximum 71%, Figure 5), while patients progressed through difficulty levels for each exercise. Despite this good average performance, it should be noted that the variability was quite high (on average ±21%), indicating that some of the exercises were not as well adapted to the patients as others. Figure 5 further illustrates this and shows the performance and difficulty level progression of each exercise for one representative patient (P4).

**Figure 6:**
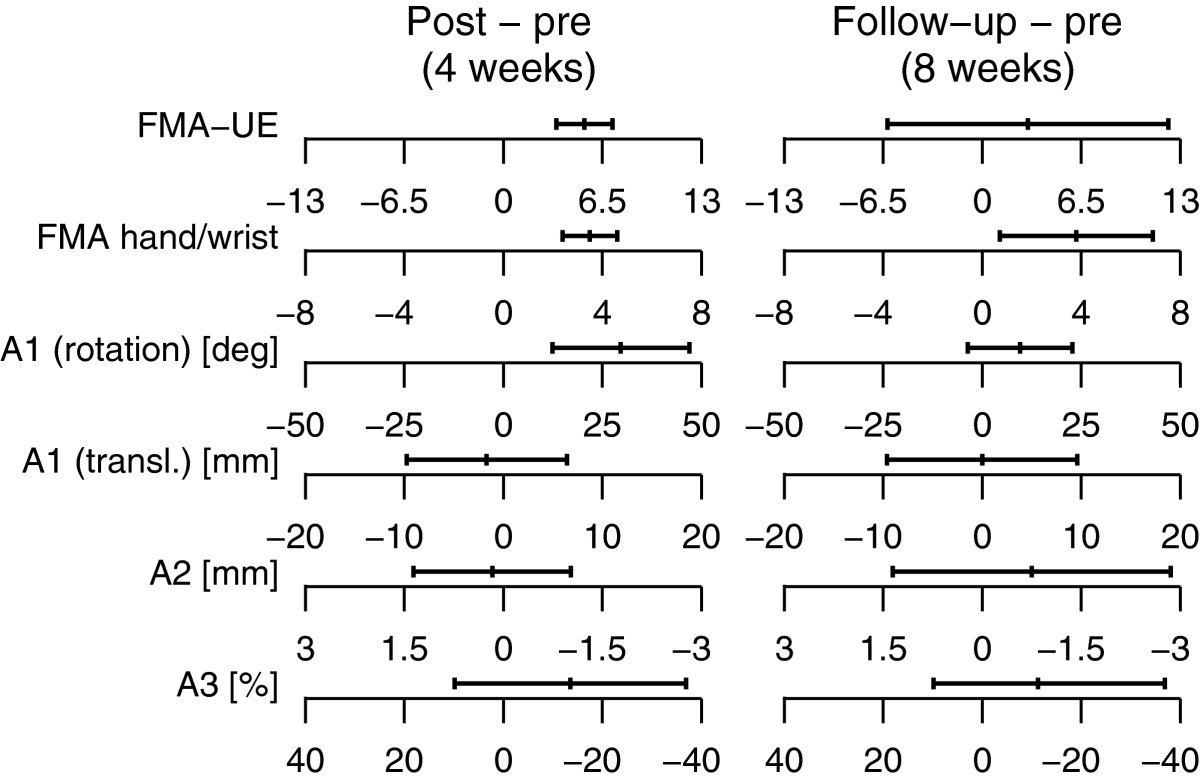
**Improvement in clinical and robotic assessment scores.** mean and 95% confidence interval of the change in the clinical (total FMA-UE score and FMA hand/wrist subscore) and robotic assessment scores (rotational and translational range of motion, proprioception (A2) and haptic perception (A3)) from *pre* to *post* assessment (left panel) and from the *pre* to *follow-up* assessment (right panel). Positive changes (negative changes in the case of A2 and A3) indicate an improvement on the assessment scale, i.e. an impairment reduction.

**Figure 7:**
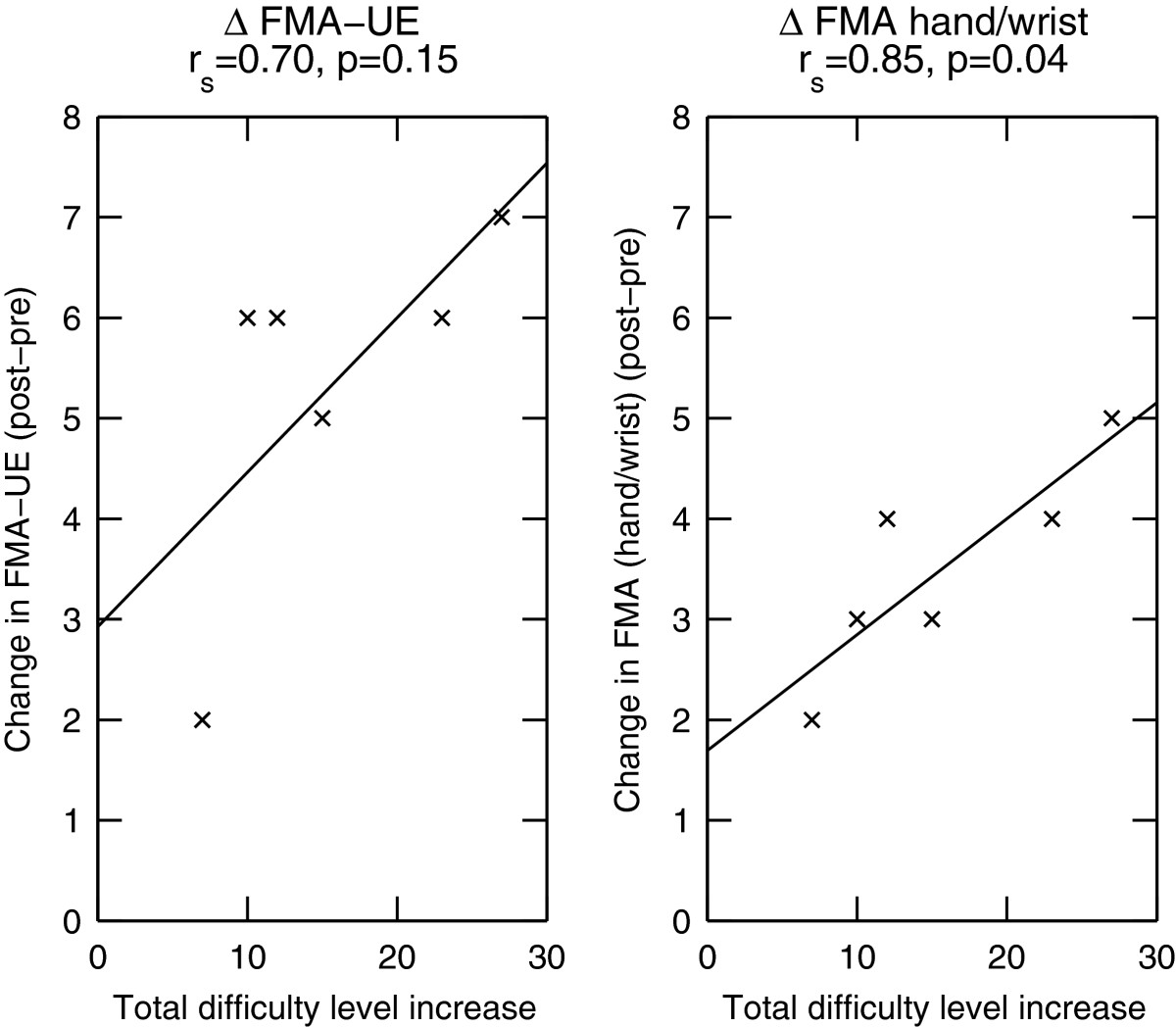
**Difficulty level increase correlates with clinical scores.** The difficulty level improvement summed over all 7 exercises correlates with the changes in the FMA-UE (total score and hand/wrist subscore) from the *pre* to the *post* assessment. *r*_*s*_ is the Spearman’s rank correlation coefficient. The line through the data points was fitted by linear regression.

Both robotic and clinical assessments showed improvements over the course of the study (Figure 6). On average, patients showed an improvement on the *ROM*_*φ*_ (*pre* to *post*: +29.7°, *pre* to *follow-up*: +9.6°), maintained their baseline assessment performance on the *ROM*_*x*_ (*pre* to *post*: -1.7 mm, *pre* to *follow-up*: 0 mm), improved their compliance perception (*pre* to *post*: -13.5%, *pre* to *follow-up*: -11.3%) while proprioception initially worsened slightly but eventually improved (*pre* to *post*: +0.17 mm, *pre* to *follow-up*: -0.8 mm). A mean improvement in the total FMA-UE score of 5.3 points (*pre* to *post*) and 3.0 points (*pre* to *follow-up*) was observed, with an improvement of the hand/wrist subscore of the FMA-UE of 3.5 points (*pre* to *post*) and 3.8 points (*pre* to *follow-up*). Note that the larger improvement in the hand/wrist subscore than in the overall FMA score at *follow-up* is mainly explained by one patient who showed a decrease of 10 points in FMA at *follow-up*. A correlation (*r*_*s*_= 0.85, p = 0.04, Spearman’s rank correlation) was found between the change in FMA-UE subscore for the hand/wrist and the number of difficulty levels that were progressed over all exercises (*Σ*(*L*_*i*,*final*_), *i* ∈ {1,7}). Similarly, a correlation was observed between *Σ*(*L*_*i*,*final*_) and the total FMA-UE score (*r*_*s*_= 0.70, p = 0.15, Spearman’s rank correlation) (Figure 7).

## Discussion

In this paper we presented a novel assessment-driven method to select and adapt difficulty levels in robot-assisted therapy. The proposed approach combines initial robotic assessments to select patient-specific therapy levels adapted to the individual’s deficits, with a simple automatic adaptation routine allowing patients to progress through these levels based on their performance, as objectively measured by the robot. A proof of concept was implemented in the context of a 4-week pilot study focusing on rehabilitation of hand function with 6 subacute stroke patients.

### The need for patient-specific difficulty levels

Stroke survivors can be highly heterogeneous in terms of sensorimotor and cognitive impairments, as well as in their prognosis for recovery [39]. In the present study, despite presenting similar initial FMA-UE scores (56.0 ±3.7) at baseline, stroke patients showed substantial differences in proprioception and haptic perception as revealed by the robotic assessments using the ReHapticKnob. First, this underlines that the FMA-UE scale, often used to evaluate upper limb motor impairment, does not capture hand sensorimotor deficits well [40]. Secondly, the robotic assessments focusing on sensory perception highlight that, despite exhibiting rather mild motor deficits, most of the participants still suffered from sensory impairments. Sensory impairment is often not a focus in rehabilitation [41], despite growing evidence of its importance for motor learning and recovery [42]. In this sense, the proposed neurocognitive approach to robot-assisted hand rehabilitation, with its primary goal of perceiving and interpreting sensory information from the impaired limb, proposes a novel way to assess and rehabilitate hand function. It is also interesting to note that the psychophysics methods used in the robotic assessments to evaluate sensory thresholds converged in most of the cases, leading to assessment sessions of short duration (typically below 15 minutes per assessment), underlining the feasibility of such an approach in clinical routine. An initial assessment provides objective values that serve as baseline and allow to track functional changes from the very beginning of the therapy. However, a validation of the proposed robotic assessments in a larger population is necessary to test their validity and reliability.

Altogether, results of the robotic assessments illustrate that, in order to confront patients with an appropriate level of challenge from the beginning of the first therapy session, individualized levels of difficulty for each neurocognitive exercise are needed. It was shown by other groups that, in the absence of such an initial assessment-based difficulty selection, a large number of therapy trials or sessions may be needed to reach challenging difficulty levels, even with fast adapting difficulty modulation algorithms. In their study with the ADAPT system, an end-effector presenting different real-life objects to manipulate against various resistance levels, Choi and colleagues showed that on average 30 trials were needed for chronic stroke patients to reach a challenging difficulty level [6]. In a robot-assisted framework where therapy exercises are composed of a large number of repetitions (i.e. over 100), this time for adaptation is likely not an issue. However, such a delay to reach challenging exercise parameters is not suitable in the context of neurocognitive exercises, where the therapy goal is to focus on movement quality, and the cognitive processing of perceived sensory information results in a lower number of therapy trials per session, which can be as low as 20 trials per exercise and session [28].

### Control of patients’ performance level

The primary objective of this work was to present stroke patients with rehabilitation exercises that are neither too simple nor too difficult, as this is expected to maximize active participation and motivation for training while minimizing frustration, three aspects that are commonly recognized as being critical for the success of a rehabilitation intervention [5, 43]. In the literature, various types of algorithms have been tested for online decision making to modify task parameters of a robot-assisted rehabilitation exercise and modulate its difficulty. As each algorithm has its own specific advantages related the platform on which it is implemented and on the type of data available to evaluate patient performance, there is no obvious golden standard for online difficulty adaptation. Several groups developed sophisticated methods, such as partially observable Markov decision processes [23], or state-space models of recovery where the evolution of a combination of kinematic parameters is tracked to adapt exercise difficulty when parameters reach plateau performance [11, 19]. Other groups used update functions with a variable forgetting factor, computed based on performance in previous trials [6], or based on the evolution of kinematic or physiological parameters [13]. Simpler approaches consist in comparing the performance achieved in a block of trials to a target performance defined by the therapist prior to the session and adapted to the patient’s impairment level, or determined based on the patient’s prior performance in the specific exercise [20, 22]. The rate at which the adaptation of difficulty is achieved also varies widely in the literature, with algorithms adapting difficulty on a trial per trial basis [6, 13], per blocks of a few trials [21, 22], or at the beginning of a session based on the performance of the previous session [11, 19, 23].

While all of the proposed methods for therapy adaptation reported good ability to modulate difficulty, we chose to implement a therapy adaptation approach based on patient-specific levels of difficulty, in which progression from level to level is based solely on the percentage of successful trials achieved during the previous session, i.e. performance in each exercise. This criterion is easy to understand by patients and therapists as opposed to algorithms based on abstract parameter optimization, and could contribute to maximizing engagement and motivation. Also, this type of algorithm was shown to be well-accepted and to provide good results in clinical application [21, 44]. In our algorithm, a target performance value of 70% was selected. This choice was motivated based on our results from prior studies with stroke patients [24, 25], as well as by values reported in literature [45].

Thanks to the initial difficulty adjustment, patients directly started with an average success rate close to the targeted 70% (62% averaged over all exercises). This underlines that from the first few trials, patients were properly challenged during the therapy session, and could appropriately engage in the task. Over the course of the 15 therapy sessions, the proposed difficulty adaptation algorithm maintained patients’ average performance close to the targeted 70% (within the range of [57,71]%) by automatically increasing levels of difficulty according to measured performance. As our approach personalizes the rate of difficulty increase in the levels of each exercise based on the initial results of robotic assessments (*ROM*_*φ*_, *DL* and *Wf*), we ensure that the ability of patients to improve in levels of difficulty is not influenced by the initial level of impairment.

The achieved degree of control over session performance throughout the course of the therapy is in line with the results of Choi and colleagues, who observed, on average, a variation of 33% in success rate around the challenge point identified by their algorithm [6].

### Reduction in hand and arm impairment

Over all exercises, patients improved between 7 and 27 difficulty levels during the course of the 4 weeks of therapy. This progression could be attributed partly to familiarization with the robot and exercises, and to a reduction in upper limb impairment. While these two factors are difficult to decouple, we observed that the number of levels progressed by patients was correlated with improvements on the FMA-UE, and especially the FMA-UE subscore for the hand/wrist (Figure 7). These correlations suggest that an increased performance in the exercises (increase in difficulty levels) does not simply correspond to a learning of exercise mechanisms (e.g. elaborating a strategy to better achieve the task), but that the proposed therapy led to a decrease in impairment. Impairment was found to be reduced especially at the level of the hand and wrist, where the interaction with the robot takes place, but also at the level of the proximal part of the arm, in line with results of previous work [25, 46].

Comparison of robotic measures between the *pre* and *post* assessments further supports these conclusions, with patients performing better (i.e. showing smaller minimal detectable differences) after the end of therapy, suggesting improved hand sensory function. This is an important result, as the implemented neurocognitive exercises specifically focused on training sensory perception. Difficulty increments between levels were designed to present sensory stimuli close to the patient’s sensory thresholds measured during the initial assessments.

The correlations between clinical scores and progression in difficulty levels suggest that the latter could be seen as an indirect way to monitor recovery on a daily basis, without the need to perform additional time-consuming clinical or robotic assessments.

### Limitations of this pilot study

Despite promising results for the control of overall performance at the group level, a relatively large performance variability could be observed. This was due to some of the neurocognitive exercises being over- or under-challenging for patients, and thus requires improvement for future studies. For example, the initial parameters of exercise *E*_2_ were pre-defined and not adjusted to the ability of the patient as measured in the robotic *pre* assessment. This resulted, at least initially, in exercises that were too simple for some participants, as shown for the representative subject P4 (Figure 5). In return, exercises *E*_5_ and *E*_6_ (sensorimotor memory) were found to be overly difficult (i.e. average performance below 47%) for most patients, due to the small error band allowed for the reproduction of movements, which made the task too demanding. Nevertheless, the initial adaptation of the exercise parameters resulted in an average performance close to the desired 70% level.

Another limitation of the present study lies in the relatively low number and limited range of initial motor impairment of patients that could be recruited for this pilot study (between 52 and 61 on the FMA-UE scale). It should nevertheless be noted that the proposed neurocognitive exercises were also designed to allow patients with more severe motor impairment to actively engage in robot-assisted therapy, as shown in our previous work [28].

## Conclusions

The results of this pilot study suggest that robotic assessments of hand sensorimotor function can be used to tailor robot-assisted therapy parameters to the ability of each individual patient. This allows to optimally balance exercise difficulty from therapy onset. Further, automatic and progressive modulation of therapy difficulty assures that patients perform at a success level that should keep the therapy engaging, rewarding and motivating. While the proposed concept of patient-tailored and adaptive robot-assisted rehabilitation was evaluated in the context of a pilot study on neurocognitive robot-assisted rehabilitation of hand function, it is generalizable to other robotic platforms and limb segments using robotic assessments and adaptation parameters specific to the capabilities of the platform. This approach further has the potential to impact the design and implementation of future therapeutic protocols for unsupervised therapy, both in the clinic and the home environment.

## Authors’ original submitted files for images

Below are the links to the authors’ original submitted files for images. Authors’ original file for figure 1 Authors’ original file for figure 2 Authors’ original file for figure 3 Authors’ original file for figure 4 Authors’ original file for figure 5 Authors’ original file for figure 6 Authors’ original file for figure 7

## Abbreviations

FMA-UE: Fugl-Meyer assessment of the upper extremity
DOF: Degrees-of-freedom
DL: Difference limen
ROM: Range of motion
2AFC: Two-alternative forced choice
PEST: Parameter estimation by sequential testing
Wf: Weber fraction.
