# Supplementary figures and images for: Assessment-driven selection and adaptation of exercise difficulty in robot-assisted therapy: a pilot study with a hand rehabilitation robot
Source: J Neuroeng Rehabil. 2014 Nov 15;11:154. doi: 10.1186/1743-0003-11-154 (PMC4273449; doi:10.1186/1743-0003-11-154)

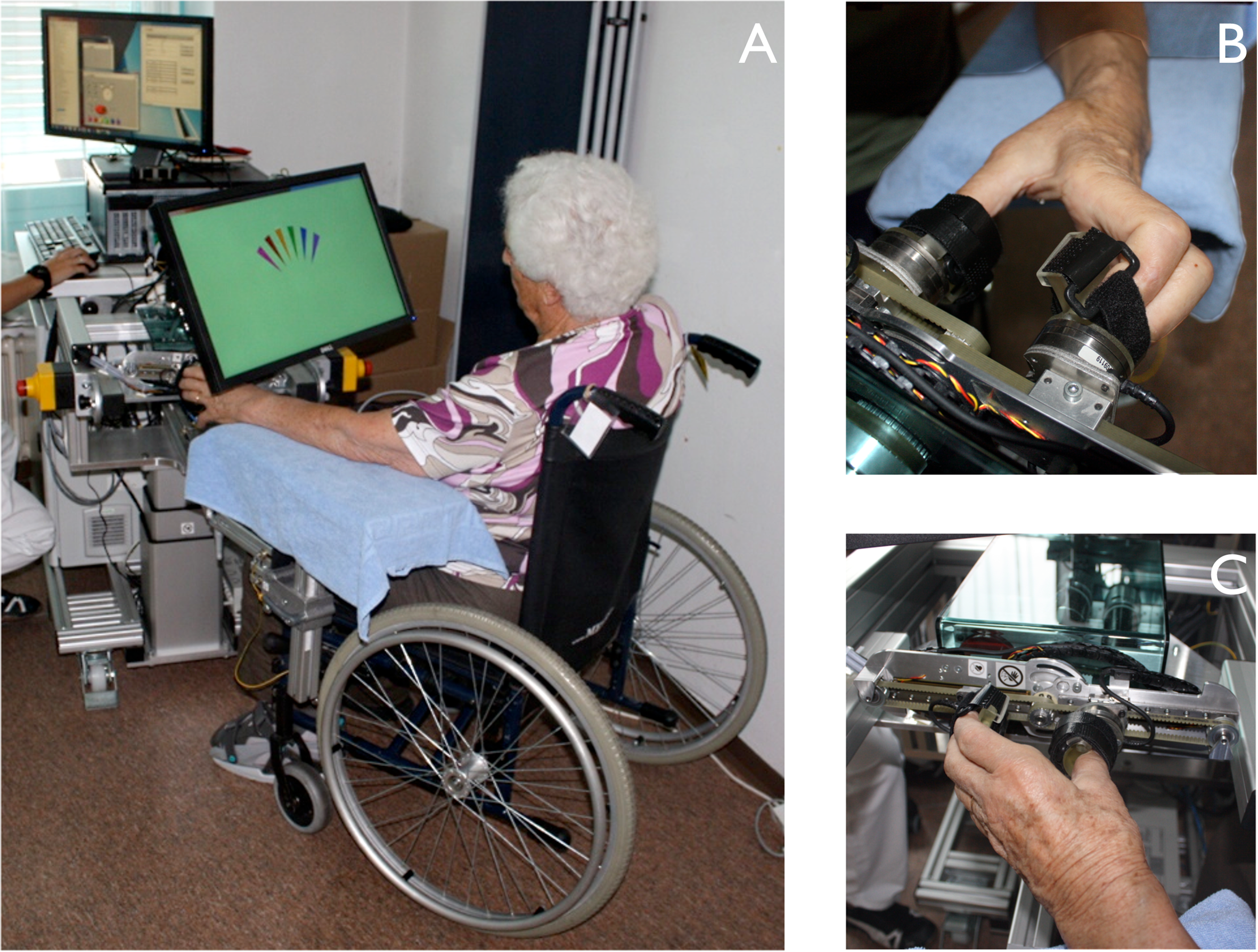

Supplement: Supplementary file 1 — Authors’ original file for figure 1 [file 12984_2014_681_MOESM1_ESM.tif]

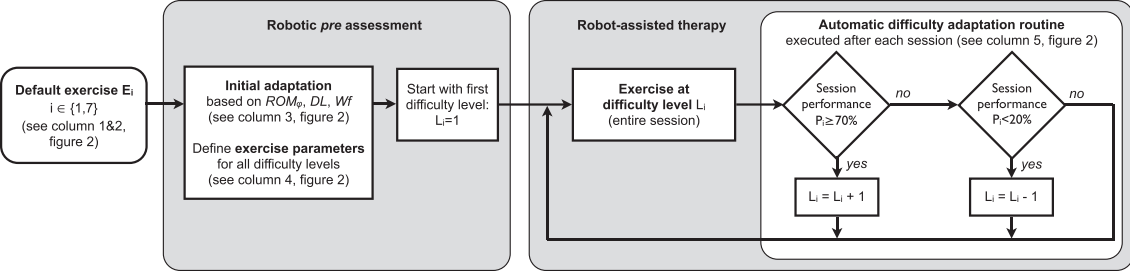

Supplement: Supplementary file 3 — Authors’ original file for figure 3 [file 12984_2014_681_MOESM3_ESM.pdf]

Group level (mean  $\pm$  std)

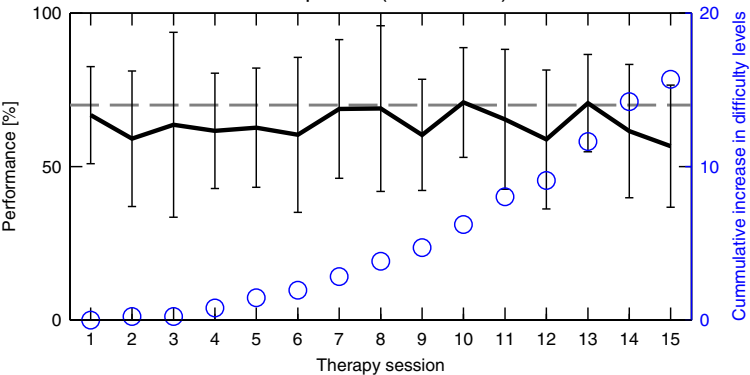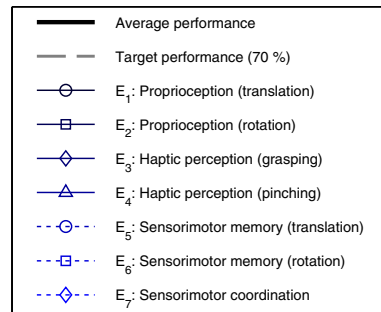

Patient P4

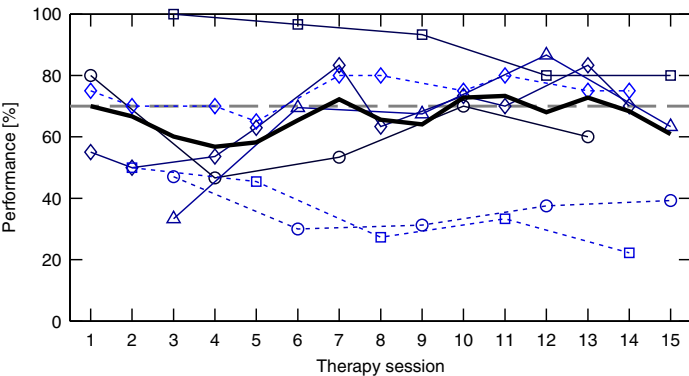

Patient P4

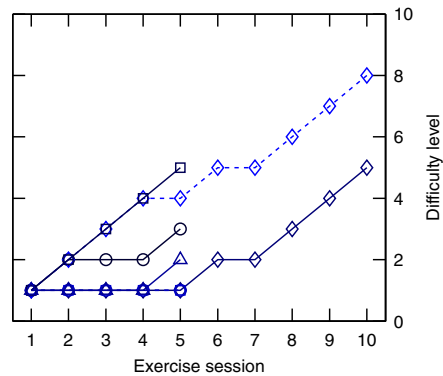

Supplement: Supplementary file 5 — Authors’ original file for figure 5 [file 12984_2014_681_MOESM5_ESM.pdf]

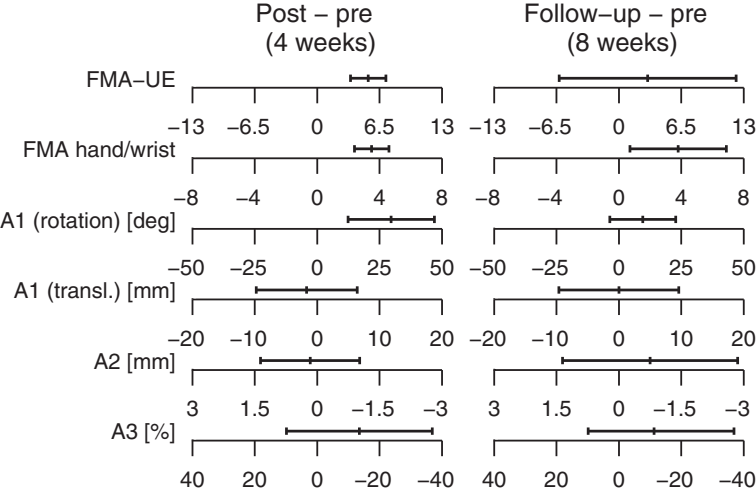

Supplement: Supplementary file 6 — Authors’ original file for figure 6 [file 12984_2014_681_MOESM6_ESM.pdf]

$\Delta$  FMA-UE  
 $r_s=0.70, p=0.15$

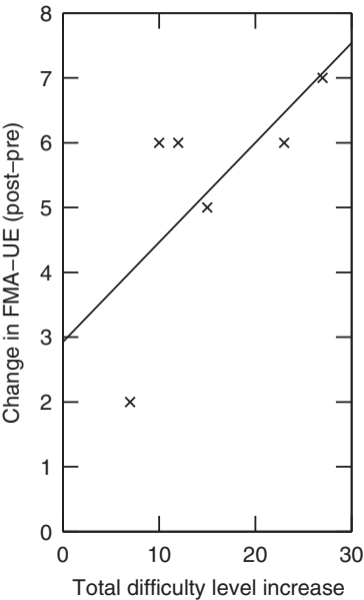

$\Delta$  FMA hand/wrist  
 $r_s=0.85, p=0.04$

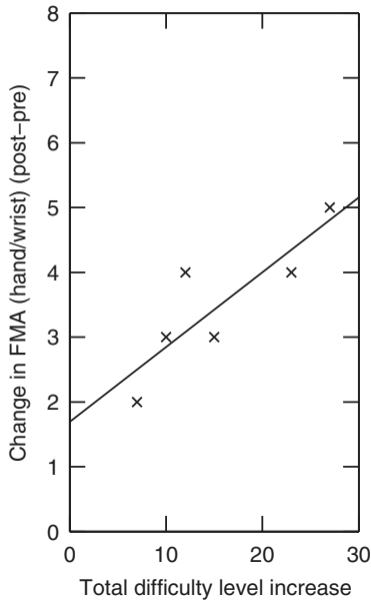

Supplement: Supplementary file 7 — Authors’ original file for figure 7 [file 12984_2014_681_MOESM7_ESM.pdf]
