# Supplementary material for: Assessment-driven selection and adaptation of exercise difficulty in robot-assisted therapy: a pilot study with a hand rehabilitation robot
Source: J Neuroeng Rehabil. 2014 Nov 15;11:154. doi: 10.1186/1743-0003-11-154 (PMC4273449; doi:10.1186/1743-0003-11-154)

Exercise description	Visual feedback	Initial adaptation	Exercise parameters	Performance metric
**E_1: Proprioception**<br>(Passive grip aperture identification)<br><br>The robot closes the hand from an initial grasping aperture d_i (adjustable in the range [102, 122] mm based on hand size) to one out of N target apertures. The N target apertures differ by δ_d and are centered around d_i-22 mm.	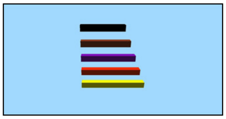<br>N sticks indicate the N grasping apertures. *Identification feedback*: a green check mark (correct ans.) or a red cross (wrong ans.) is displayed next to the correct (target) stick.	The difference between target apertures is a function of the assessed distance *DL*: δ_d = *f*(distance *DL*)<br><br>The assessed distance *DL* (assessment A_2) is limited to the range [2,10] mm	5 difficulty levels:<br><br>Number of target apertures<br>N = {3,4,5,5,5}<br><br>Target aperture difference<br>δ_d = {2,1.6,1.2,1.1,1} × (distance *DL*)	Percentage of correct identification trials
**E_2: Proprioception**<br>(Passive pronosupination angle identification)<br><br>The robot rotates the hand from an initial angle (-60°, i.e. counterclockwise) to one out of N target angles. The N target angles differ by δ_φ and are centered around 0°.	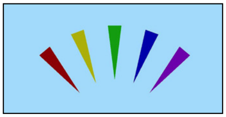<br>N triangles indicate the N target angles. *Identification feedback*: a green edge (correct ans.) or a red edge (wrong ans.) is displayed around the correct (target) angle.	none	5 difficulty levels:<br><br>Number of target apertures<br>N = {3,4,5,6,7}<br><br>Target aperture difference<br>δ_φ = {30,25,20,15,10}°	Percentage of correct identification trials
**E_3: Haptic perception**<br>(Stiffness identification during grasping)<br><br>The robot renders N sponges (spring-damper combinations) which have to be identified based on their viscoelastic resistance during squeezing. Rendered stiffness and damping pairs vary by η percent from one to another and are centered around K_{medium} = 550 N/m and B_{medium} = 35 N/(m/s).	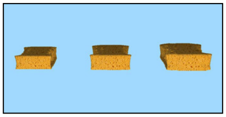<br>All N sponges are displayed and animated during squeezing. *Identification feedback*: the rendered sponge is colored green (correct ans.) or red (wrong ans.).	The relative difference between the visco-elasticities is a function of the assessed stiffness *Wf*: η = *f*(stiffness *Wf*)<br><br>The assessed stiffness *Wf* (assessment A_3) is limited to the range [7.5, 45] %	10 difficulty levels:<br><br>Number of sponges<br>N = {3,3,4,4,5,5,5,5,5,5}<br><br>Relative difference between viscoelasticities<br>η = {2,1.9,1.8,1.7,1.6,1.5, 1.4,1.3,1.2,1.1} × (stiffness *Wf*)	Percentage of correct identification trials
**E_4: Haptic perception**<br>(Stiffness identification during pinching)<br><br>The robot renders N springs (spring-damper combinations) which have to be identified based on their viscoelastic resistance during vertical index finger pinching. Rendered stiffness and damping pairs vary by η percent from one to another and are centered around K_{medium} = 300 N/m and B_{medium} = 20 N/(m/s).	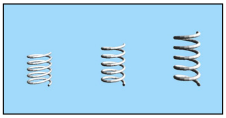<br>All N springs are displayed and animated when compressed. *Identification feedback*: the rendered spring is colored green (correct ans.) or red (wrong ans.).	The relative difference between the visco-elasticities is a function of the assessed stiffness *Wf*: η = *f*(stiffness *Wf*)<br><br>The assessed stiffness *Wf* (assessment A_3) is limited to the range [7.5, 45] %	5 difficulty levels:<br><br>Number of springs<br>N = {3,4,5,5,5}<br><br>Relative difference between viscoelasticities<br>η = {2,1.8,1.6,1.4,1.2} × (stiffness *Wf*)	Percentage of correct identification trials
**E_5: Sensorimotor memory** (Grip aperture)<br><br>*Teach*: the robot closes the hand from an initial grasping aperture d_i (adjustable in the range [102, 122] mm based on the hand size) to a randomly selected target grasping aperture d_t in the range [70, d_i-2] mm. After 2 seconds the hand is moved back to d_i.<br>*Reproduce*: the patient is asked to move to the „taught" target grasping aperture and hold this position for 2 seconds (position logging). A trial is correct if the logged position lies within the error band [d_t-δ_e/2, d_t+δ_e/2]. A damped force field helps to smoothen the movement of the patient: B=β × 50 N/(m/s).	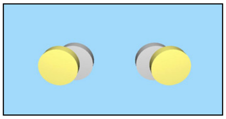<br>No visual feedback is provided during „teach" and „reproduce" phases.<br>After trial completion a green check mark is shown if the trial was correct.<br>Additionally, the target position is shown in grey and the registered position in yellow (or in red if the trial was wrong).	The error band is a function of the assessed distance *DL*: δ_e = *f*(distance *DL*)<br><br>The assessed distance *DL* (assessment A_2) is limited to the range [2,10] mm	5 difficulty levels:<br><br>Error band<br>δ_e = {1.2,1.1,1,0.9,0.8} × (distance *DL*)<br><br>Reduction of damping support:<br>β={1,0.75,0.5, 0.25,0}	Percentage of correct reproduced trials
**E_6: Sensorimotor memory**<br>(Pronosupination angle)<br><br>*Teach*: the robot rotates the forearm from an initial angle (-60°, i.e. counter-clockwise) to a target angle φ_t randomly selected from a range R. After 2 seconds the hand is returned to φ_i.<br>*Reproduce*: the patient is asked to rotate to the „taught" target angle and hold this angle for 2 seconds (angle logging). A trials is correct if the logged angle lies within the error band: [φ_t-δ_e/2, φ_t+δ_e/2]. A damped force field helps to smoothen the movement of the patient: B=β × 50 Nm/(°/s).	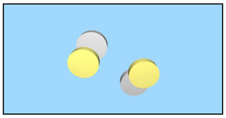<br>No visual feedback is provided during „teach" and „reproduce" phases.<br>After trial completion a green check mark indicates if the trial was correct.<br>Additionally, the target angle is shown in grey and the logged angle in yellow (or in red if the trial was wrong).	Assessed rotational ROM_φ (assessment A_1) defines the range R from which the target angle is randomly selected: R=*f*(ROM_φ)<br><br>The range R is limited to [-60, 60] °	5 difficulty levels:<br><br>Range from which φ_t is randomly selected<br>R = {1,1.05,1.1,1.15,1.2} × ROM_φ<br><br>Reduction of damping support:<br>β={1,0.75,0.5, 0.25,0}<br><br>Error band<br>δ_e = {10,8,6,4,2} °	Percentage of correct reproduced trials
**E_7: Sensorimotor coordination**<br>(Haptically cued forearm rotation)<br><br>The patient is asked to explore the rotational DOF in order to find a target angle φ_t which is indicated haptically by means of a small haptic valley/gap with amplitude *A* along the translational DOF. The robot has to be held in [φ_t-2°, φ_t+2°] for 2 seconds to register and verify the correctness of the current robot angle φ. A rotational damping field with damping constant B smooths the patient's movement. A trial is successful when the target angle is found within 60 seconds. Otherwise the robot moves the patient passively to φ_t.	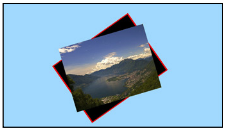<br>A rotating picture reflects the current robot angle φ. A green frame is drawn around the picture when the target angle has been found successfully. Only during task familiarization the target angle φ_t is visualized by a black square.	Assessed rotational ROM_φ (assessment A_1) defines the range R from which the target angle is randomly selected: R=*f*(ROM_φ)<br><br>The range R is limited to [-60, 60] °	10 difficulty levels:<br><br>Applicable range of target angles:<br>R = {1,1.02,1.04,1.06,1.08, 1.1,1.12,1.14,1.16,1.18} × ROM_φ<br><br>Reduction of damping support:<br>B={0.9,0.8,0.7,0.6, 0.5,0.4,0.3,0.2,0.1,0} e-3 [Nm/(°/s)]<br><br>Haptic valley amplitude:<br>*A*={1.8,1.65,1.5,1.35,1.2,1.05,0.9, 0.75,0.6,0.45} [mm]	Number of successful trials within exercise time.<br>20 trials (or more) in 15 min corresponds to 100%.

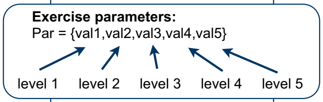

Supplement: Supplementary file 2 — Authors’ original file for figure 2 [file 12984_2014_681_MOESM2_ESM.pdf]
