# Supplementary material for: Assessment-driven selection and adaptation of exercise difficulty in robot-assisted therapy: a pilot study with a hand rehabilitation robot
Source: J Neuroeng Rehabil. 2014 Nov 15;11:154. doi: 10.1186/1743-0003-11-154 (PMC4273449; doi:10.1186/1743-0003-11-154)
Supplement: Supplementary file 4 — Authors’ original file for figure 4 [file 12984_2014_681_MOESM4_ESM.pdf]

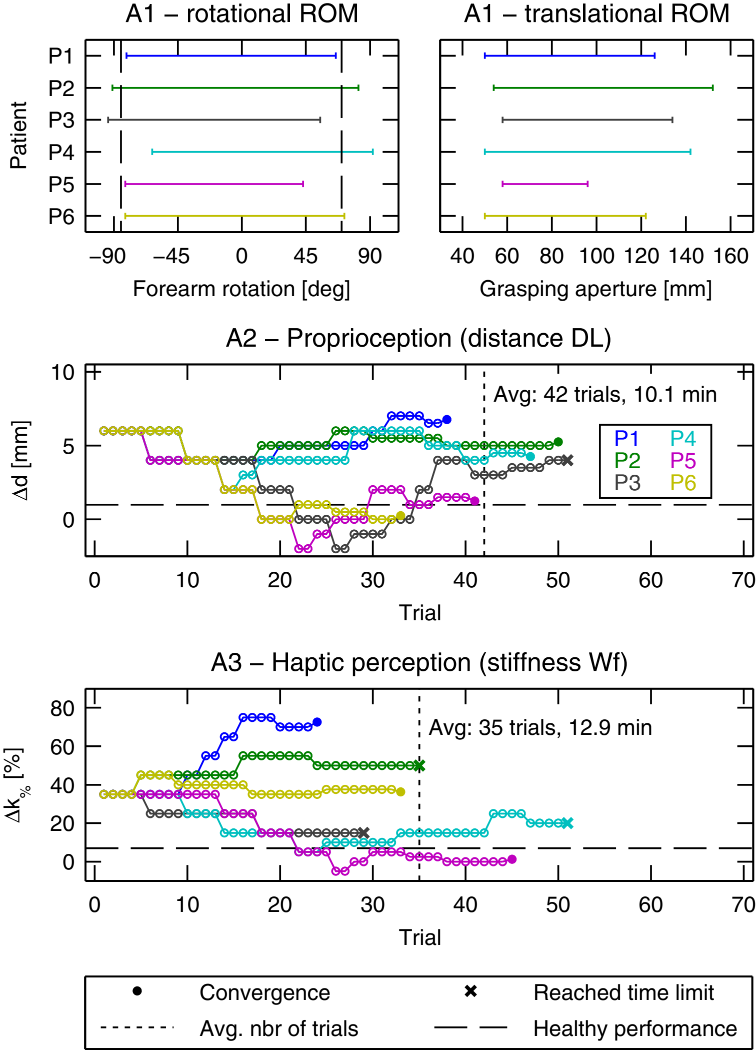

A1 – rotational ROM
A1 – translational ROM
Patient
P1
P2
P3
P4
P5
P6
–90
–45
0
45
90
Forearm rotation [deg]
40
60
80
100
120
140
160
Grasping aperture [mm]
A2 – Proprioception (distance DL)
Δd [mm]
Avg: 42 trials, 10.1 min
Trial
A3 – Haptic perception (stiffness Wf)
Δk% [%]
Avg: 35 trials, 12.9 min
Trial
Convergence
Reached time limit
Avg. nbr of trials
Healthy performance
